# Associations between early tumor shrinkage/depth of response and survival from the ARCAD database

**DOI:** 10.1093/jncics/pkaf042

**Published:** 2025-04-25

**Authors:** Hideaki Bando, Yuriko Takeda, Toshihiro Misumi, Tomomi Nishikawa, Masashi Wakabayashi, Kentaro Yamazaki, Eiji Oki, Jean-Yves Douillard, Cornelis J A Punt, Miriam Koopman, Eric Van Cutsem, Carsten Bokemeyer, Alan P Venook, Heinz-Josef Lenz, Yoshihiko Maehara, Thierry Andre, Qian Shi, Aimery de Gramont, Takayuki Yoshino

**Affiliations:** Department of Gastroenterology and Gastrointestinal Oncology, National Cancer Center Hospital East, Chiba, Japan; Department of Data Science, National Cancer Center Hospital East, Chiba, Japan; Department of Data Science, National Cancer Center Hospital East, Chiba, Japan; Department of Data Science, National Cancer Center Hospital East, Chiba, Japan; Department of Data Science, National Cancer Center Hospital East, Chiba, Japan; Department of Data Science, National Cancer Center Hospital East, Chiba, Japan; Division of Gastrointestinal Oncology, Shizuoka Cancer Center, Shizuoka, Japan; Department of Surgery and Science, Graduate School of Medical Sciences, Kyushu University, Fukuoka, Japan; Medical Oncology Department, Integrated Centers for Oncology Nantes, Nantes, France; Department of Epidemiology, Julius Center for Health Sciences and Primary Care, University Medical Centre, Utrecht, The Netherlands; Department of Epidemiology, Julius Center for Health Sciences and Primary Care, University Medical Centre, Utrecht, The Netherlands; Department of Gastrointestinal and Liver Diseases, Digestive Oncology Unit, University Hospital Gasthuisberg and University of Leuven (KUL), Leuven, Belgium; Department of Oncology, Hematology and Bone Marrow Transplantation With Section of Pneumology, University Medical Center Hamburg-Eppendorf, Hamburg, Germany; Department of Medicine, University of California San Francisco, San Francisco, CA, United States; Department of Gastrointestinal Oncology, Keck School of Medicine at USC, Los Angeles, CA, United States; Kyushu Central Hospital of the Mutual Aid Association of Public School Teachers, Fukuoka, Japan; Department of Medical Oncology, Sorbonne University, Hospital Saint Antoine, Paris, France; Department of Quantitative Science Research, Mayo Clinic, Rochester, MN, United States; Department of Medical Oncology, Franco-British Institute, Levallois-Perret, France; Department of Gastroenterology and Gastrointestinal Oncology, National Cancer Center Hospital East, Chiba, Japan; Department of Data Science, National Cancer Center Hospital East, Chiba, Japan

## Abstract

**Background:**

Early tumor shrinkage and depth of response have emerged as potential prognostic indicators in metastatic colorectal cancer (CRC). However, their associations with overall survival, progression-free survival (PFS), and postprogression survival in patients receiving anti–epidermal growth factor receptor (EGFR) antibodies or bevacizumab remain unclear.

**Methods:**

We analyzed 3219 treatment-naive patients with *RAS* wild-type metastatic CRC from 8 randomized studies (CRYSTAL, OPUS, PRIME, CAIRO2, CALGB80405, WJOG4407G, ATOM, PARADIGM) in the Aid and Research in Digestive Cancerology database. Early tumor shrinkage was defined as a 20% or more reduction in tumor size at 8 ± 2 weeks, whereas depth of response was assessed by maximum tumor shrinkage at nadir. Cox regression models evaluated the associations of early tumor shrinkage and depth of response with overall survival, PFS, and postprogression survival, adjusting for confounders. A 2-sided test was conducted with a significance level of .05.

**Results:**

Early tumor shrinkage and depth of response substantially stratified overall survival, PFS, and postprogression survival outcomes across all treatment groups. Early tumor shrinkage positivity was associated with improved overall survival, PFS, and postprogression survival in anti-EGFR and bevacizumab-based therapies, with a trend toward better outcomes in the anti-EGFR group. The depth of response analysis revealed optimal cutoff values of 0.55 for anti-EGFR–based therapy and 0.47 for bevacizumab-based therapy to achieve a median overall survival of approximately 32 months.

**Conclusions:**

Early tumor shrinkage and depth of response serve as valuable prognostic markers in *RAS* wild-type metastatic CRC, particularly for patients treated with anti-EGFR antibodies. These findings highlight the potential role of early tumor shrinkage and depth of response in guiding treatment strategies and improving outcomes for patients with CRC.

## Introduction

In 2020, colorectal cancer (CRC) ranked as the third-most prevalent cancer worldwide in terms of new cases, with 1 148 515 cases diagnosed in the colon and 732 210 cases diagnosed in the rectum. In addition, CRC was the second-leading cause of cancer-related deaths globally, with 935 000 deaths attributed to colon cancer and 339 022 to rectal cancer.^1^

According to guidelines from the European Society for Medical Oncology, the adapted European Society for Medical Oncology Pan-Asian guidelines, and the American Society of Clinical Oncology, the preferred therapy for patients with *KRAS*, *NRAS*, and *BRAF* wild-type left-sided metastatic CRC involves combining 5-fluorouracil and leucovorin with oxaliplatin (FOLFOX) or irinotecan (FOLFIRI), alongside an anti–epidermal growth factor receptor (EGFR) antibody (cetuximab or panitumumab).^2-4^ This recommendation is based on a meta-analysis of 6 randomized trials.^5^ Furthermore, the Japanese PARADIGM trial demonstrated the superiority of first-line modified FOLFOX6 plus panitumumab over modified FOLFOX6 plus bevacizumab in populations with *KRAS*/*NRAS* wild-type left-sided primary tumors, establishing the use of first-line FOLFOX plus an anti-EGFR antibody in this subgroup.^6^ For patients presenting with *KRAS* and *NRAS* mutations; *BRAF* V600E mutations; or *KRAS, NRAS*, and *BRAF* wild-type right-sided disease, the preferred therapy is a doublet (eg, FOLFOX or FOLFIRI or oxaliplatin plus capecitabine) or triplet (oxaliplatin plus irinotecan plus 5-fuluorouracil and leucovorin) combined with anti–vascular endothelial growth factor antibody (bevacizumab).^3^^,^^7^

In CRC, the effectiveness of systemic treatments is typically assessed using objective response rate, progression-free survival (PFS), and overall survival. Although overall survival serves as the primary measure for evaluating the efficacy of a new therapy, PFS is considered an early endpoint and frequently used as a reliable surrogate for overall survival. However, beyond a median overall survival of 30 months and a median PFS of 10 months, the impact of postprogression treatment becomes more substantial, raising questions about the reliability of PFS as a predictor of overall survival.^8^^,^^9^ Indeed, past phase 3 trials comparing chemotherapy plus an anti-EGFR antibody with chemotherapy plus bevacizumab reproducibly demonstrated a clinically significant difference in overall survival, despite no difference being observed in PFS.^6^^,^^8^^,^^9^ Therefore, alternative surrogate endpoints for overall survival that measure the direct effect of the agents used on tumor size are currently being discussed.

Early assessment of tumor reduction may serve as a valuable tool in guiding treatment strategies during the initial restaging phase. Recently, early tumor shrinkage has garnered attention among clinicians as a promising prognostic indicator for overall survival in patients with metastatic CRC undergoing first-line therapy.^10-15^ Although early tumor shrinkage, akin to any response to anticancer therapy, represents a continuous parameter, a cutoff-based assessment has been introduced to delineate early tumor shrinkage–positive cases from early tumor shrinkage–negative cases. Although the optimal cutoff values and timing for early tumor shrinkage assessment have yet to be precisely defined, 8 weeks after initiating therapy and a tumor shrinkage of 20% or more are likely to be established, regardless of the regimen.^10-15^ In addition, depth of response, which signifies the maximum observed tumor reduction in a patient, has emerged as a continuous efficacy metric with potential implications for predicting long-term treatment outcomes.^11-14^^,^^16-18^

For CRC, individual patient data from clinical trials concerning metastatic CRC have been shared across Europe, Japan, and the United States through the Aid and Research in Digestive Cancerology (ARCAD) Foundation database project since 2006.^19^ The CRC ARCAD global database project has been established as a comprehensive repository of historical individual patient data, encompassing information from more than 43 324 patients across more than 64 studies worldwide, and it continues to expand. These trials have been sponsored by various entities, including industry, governments, and academic groups, ensuring the inclusion of high-quality clinical data.^19^^,^^20^ In this study, we evaluated the potential of early tumor shrinkage and depth of response as surrogate endpoints for overall survival and postprogression survival, defined as survival following first-line therapy, in patients treated with chemotherapy plus an anti-EGFR antibody or bevacizumab.

## Methods

### Patient selection and study cohort

From the individual patient data extracted from 64 studies within the ARCAD metastatic CRC database, we selected a cohort of treatment-naive patients diagnosed with unresectable *RAS* wild-type metastatic CRC who subsequently received either chemotherapy combined with an anti-EGFR antibody or bevacizumab or chemotherapy alone.

### Definition and assessment of early tumor shrinkage and depth of response

In this analysis, early tumor shrinkage was defined as a reduction in tumor size of 20% or more at 8 ± 2 weeks. Cases were categorized as early tumor shrinkage positive if the patients achieved a reduction of 20% or greater in the sum of the sizes of targeted lesions and as early tumor shrinkage negative if the reduction was less than 20%. Depth of response was defined as the maximum percentage reduction in targeted lesions from baseline following treatment. To validate the reliability of early tumor shrinkage and depth of response as tumor evaluation metrics, we summarize these measures based on the number of tumor lesions and the total tumor diameter.

### Statistical analysis

The impact of early tumor shrinkage positivity and negativity, depth of response, and the use of anti-EGFR antibodies and bevacizumab on overall survival, PFS, and postprogression survival was assessed using multivariate Cox regression analysis, adjusted for age, sex, and ECOG-ACRIN performance status. Depth of response was evaluated across each quantile of the maximum percentage reduction. Survival analyses were conducted using the Kaplan-Meier method to generate survival curves and estimate median survival times. In these analyses, the starting point was the date of random assignment; overall survival was defined as the time from random assignment to death from any cause, PFS was defined as the time from random assignment to disease progression or death from any cause, and postprogression survival was defined as the time from the date of progression after first-line therapy to death from any cause for patients who experienced disease progression during first-line therapy. To determine the optimal cutoff value for depth of response and its corresponding sensitivity and specificity, receiver operating characteristic curve analysis using the Youden index was performed. The hazard ratio (HR) for overall survival was calculated for each percentage cutoff using multivariate Cox regression analysis, adjusted for age, sex, and ECOG-ACRIN performance status. All analyses were prespecified, and a 2-sided test was conducted with a significance level of .05. However, no adjustments for multiplicity were made due to the exploratory nature of the study. Statistical analyses were performed using SAS, version 9.4, statistical software (SAS Institute Inc).

### Ethical approval and compliance

The clinical protocol was approved by the Institutional Review Board of the National Cancer Center Japan (Protocol No. 2021-096 and 2021-192). The study was conducted in accordance with the principles of the Declaration of Helsinki.

## Results

### Patient selection and background

**Figure 1.**
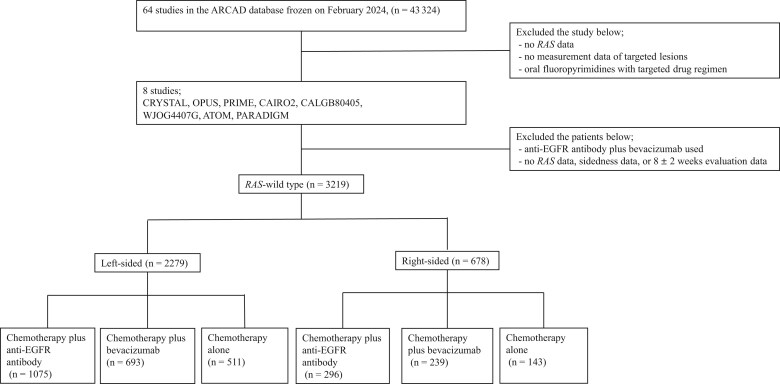
Patient selection CONSORT diagram. This diagram outlines the process of patient selection across 8 studies (CRYSTAL, OPUS, PRIME, CAIRO2, CALGB80405, WJOG4407G, ATOM, and PARADIGM) included in the ARCAD database. A total of 3219 patients with *RAS* wild-type metastatic colorectal cancer were analyzed, categorized by tumor sidedness and treatment (chemotherapy plus anti-EGFR antibody, chemotherapy plus bevacizumab, or chemotherapy alone). ARCAD = Aid and Research in Digestive Cancerology; EGFR = epidermal growth factor receptor.

We extracted individual patient data from 64 studies within the ARCAD metastatic CRC database. Patients were selected from 8 randomized trials (CRYSTAL, OPUS, PRIME, CAIRO2, CALGB80405, WJOG4407G, ATOM, and PARADIGM) that involved first-line therapy and conducted the initial tumor evaluation at 8 ± 2 weeks, excluding trials that used oral fluoropyrimidine-based treatments (Table 1). Among these cases, we excluded those without data on *RAS* status, tumor sidedness, or target lesion measurements; those whose tumor evaluations were not performed at 8 ± 2 weeks; and where the participants were treated with an anti-EGFR antibody plus bevacizumab. Finally, a total of 3219 patients with confirmed *RAS* wild-type tumors were enrolled, of whom 2279 had left-sided tumors and 678 had right-sided tumors (Figure 1).

**Table 1.** The list of clinical trials.

Study	Evaluation criteria	First assessment, wk	Study treatment	Patients randomly assigned, No.	*RAS* wild type and data on tumor location collected, No.
CRYSTAL	World Health Organization	8 ± 2	FOLFIRI + cetuximab	610	259
			FOLFIRI	611	300
OPUS	World Health Organization	8 ± 2	FOLFOX + cetuximab	173	73
			FOLFOX	171	86
PRIME (C203)	RECIST 1.0	8 ± 2	FOLFOX4	590	268
			FOLFOX4 + panitumumab	593	247
CAIRO2	RECIST 1.0	9 ± 2	oxaliplatin and capecitabine + bevacizumab	378	95
CALGB-80405	RECIST 1.0	8 ± 2	Chemotherapy + bevacizumab	897	389
			Chemotherapy + cetuximab	897	406
WJOG4407G	RECIST 1.0	8 ± 2	FOLFOX + bevacizumab	200	30
			FOLFIRI + bevacizumab	202	34
ATOM	RECIST 1.1	8 ± 2	Modified FOLFOX6 + bevacizumab	61	56
			Modified FOLFOX6 + cetuximab	61	58
PARADIGM	RECIST 1.1	8 ± 2	Modified FOLFOX6 + panitumumab	411	328
			Modified FOLFOX6 + bevacizumab	412	328

Abbreviations: FOLFIRI = 5-fluorouracil, leucovorin, and irinotecan; FOLFOX = 5-fluorouracil, leucovorin, oxaliplatin; RECIST = Response Evaluation Criteria for Solid Tumors.

Patient characteristics for patients treated with chemotherapy plus an anti-EGFR antibody and for patients treated with chemotherapy plus bevacizumab were comparable with respect to ECOG-ACRIN performance status, sex, age at enrollment, carcinoembryonic antigen levels, sum of target lesion diameters, presence of liver metastasis, number of metastatic sites, *BRAF* mutation, and surgical history. The rate of missingness was less than 5% for each variable, except for metastatic sites, carcinoembryonic antigen levels, and *BRAF* mutation, which were not collected in the original study (Tables S1 and S2).

### Summary of early tumor shrinkage and depth of response by tumor lesion count and total tumor diameter

**Figure 2.**
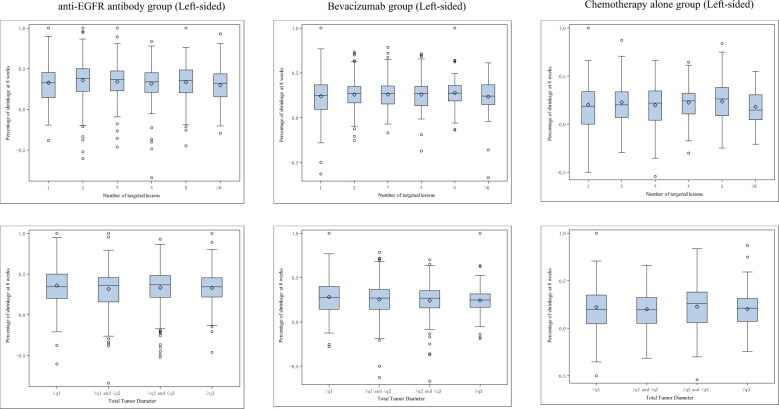
Correlations between early tumor shrinkage and baseline targeted lesions/total tumor diameter. The figure illustrates the relationship between early tumor shrinkage and the number of baseline targeted lesions or total tumor diameter. Data are presented for 3 treatment groups (anti–epidermal growth factor receptor [EGFR] antibody, bevacizumab, and chemotherapy alone), showing the percentage of tumor shrinkage at 8 weeks in left-sided tumors.

In this analysis, 766 cases were evaluated using the World Health Organization criteria^21^; 1676 cases using Response Evaluation Criteria in Solid Tumors (RECIST), version 1.0, criteria^22^; and 777 cases using RECIST, version 1.1, criteria.^23^ Given the potential for variability in the number of lesions assessed under each criterion, we first examined whether early tumor shrinkage and depth of response differed based on the number of tumor lesions or the total tumor diameter. Among patients treated with chemotherapy plus an anti-EGFR antibody (anti-EGFR antibody group), chemotherapy plus bevacizumab (bevacizumab group), and chemotherapy alone (chemotherapy-alone group), the median early tumor shrinkage was 0.3 to 0.4 in the anti-EGFR antibody group, 0.2 to 0.3 in the bevacizumab group, and 0.2 to 0.3 in the chemotherapy-alone group, regardless of the number of baseline targeted lesions or total tumor diameter (Figure 2). Furthermore, irrespective of the number of baseline targeted lesions or total tumor diameter, the median depth of response was 0.5 to 0.7 in the anti-EGFR antibody group, 0.4 to 0.5 in the bevacizumab group, and 0.4 to 0.5 in the chemotherapy-alone group (Figure S1). We examined whether early tumor shrinkage and depth of response differed based on the presence or absence of liver metastases and found that liver metastasis status did not affect the assessment of early tumor shrinkage and depth of response (Tables S3 and S4). For peritoneal dissemination and lung metastases, a high rate of missing data prevented us from conducting a valid analysis.

### Correlations between early tumor shrinkage and survival

**Figure 3.**
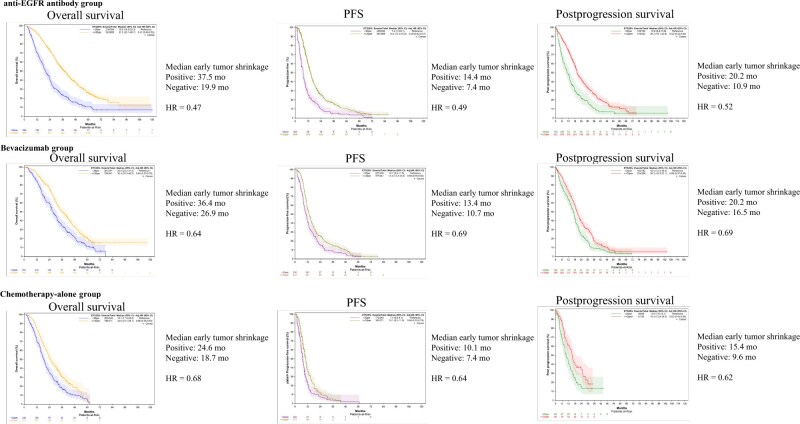
The correlations between early tumor shrinkage and survival in left-sided tumors. This figure demonstrates the correlation between early tumor shrinkage and survival outcomes (overall survival, progression-free survival [PFS], and postprogression survival) in left-sided *RAS* wild-type tumors. Early tumor shrinkage–positive patients showed improved overall survival, PFS, and postprogression survival across all treatment groups (anti–epidermal growth factor receptor [EGFR] antibody, bevacizumab, and chemotherapy alone), with hazard ratios (HRs) favoring early tumor shrinkage positivity.

To evaluate the statistical significance of achieving early tumor shrinkage, we first examined its implications in the anti-EGFR antibody group, in the bevacizumab group, and in the chemotherapy-alone group. Our findings suggest that early tumor shrinkage in patients with *RAS* wild-type metastatic CRC may serve as a prognostic marker for overall survival, PFS, and postprogression survival, irrespective of the type of targeted therapy used or the primary tumor location (Figure 3, Figure S2). Specifically, in patients with left-sided *RAS* wild-type metastatic CRC, the hazard ratios for early tumor shrinkage–positive cases compared with early tumor shrinkage–negative cases in the anti-EGFR antibody group were as follows: overall survival, 0.47 (95% confidence interval [CI] = 0.40 to 0.55); PFS, 0.49 (95% CI = 0.42 to 0.57); and postprogression survival, 0.52 (95% CI = 0.42 to 0.64). For the bevacizumab group, the hazard ratios were as follows: overall survival, 0.64 (95% CI = 0.53 to 0.76); PFS, 0.69 (95% CI = 0.59 to 0.82); and postprogression survival, 0.69 (95% CI = 0.57 to 0.85). In the chemotherapy-alone group, the hazard ratios were as follows: overall survival, 0.68 (95% CI = 0.56 to 0.83); PFS, 0.64 (95% CI = 0.52 to 0.79); and postprogression survival, 0.62 (95% CI = 0.43 to 0.90) (Figure 3). When comparing survival outcomes across different regimens, patients treated with chemotherapy plus an anti-EGFR antibody exhibited a trend toward better hazard ratios for overall survival, PFS, and postprogression survival compared with patients treated with chemotherapy plus bevacizumab or chemotherapy alone. This finding suggests a potential relationship between early tumor shrinkage and improved survival outcomes (Figure 3).

**Figure 4.**
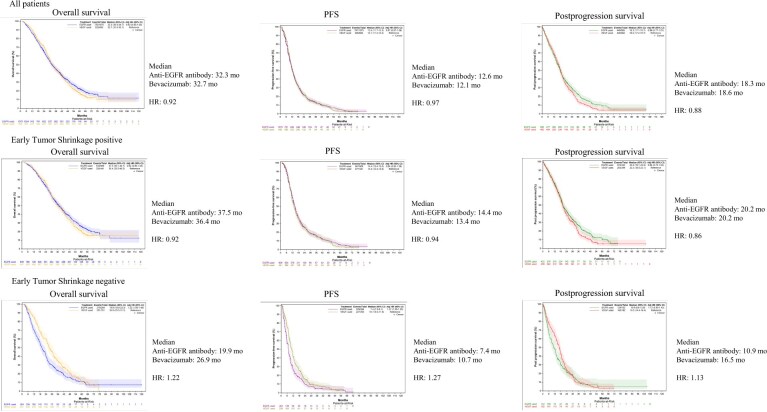
Correlations between early tumor shrinkage and treatment efficacy in left-sided tumors. The figure compares the efficacy of anti-EGFR antibody and bevacizumab treatments in early tumor shrinkage–positive and early tumor shrinkage–negative patients with left-sided tumors. Early tumor shrinkage–positive patients demonstrated similar overall survival, PFS, and postprogression survival between both treatment types, whereas early tumor shrinkage–negative patients treated with bevacizumab showed better survival outcomes. EGFR = epidermal growth factor receptor; HR = hazard ratio; PFS = progression-free survival.

Next, we compared the significance of early tumor shrinkage between patients treated with chemotherapy plus an anti-EGFR antibody and patients treated with chemotherapy plus bevacizumab. In patients with left-sided *RAS* wild-type early tumor shrinkage–positive metastatic CRC, there were no statistically significant differences in overall survival, PFS, or postprogression survival between anti-EGFR–based therapy and bevacizumab-based therapy (overall survival, HR = 0.92 [95% CI = 0.80 to 1.05]; PFS, HR = 0.94 [95% CI = 0.83 to 1.08]; postprogression survival, HR = 0.86 [95% CI = 0.73 to 1.02]). In contrast, in early tumor shrinkage–negative patients on bevacizumab-based therapy showed a trend toward better overall survival, PFS, and postprogression survival outcomes compared with patients on anti-EGFR–based therapy (overall survival, HR = 1.22 [95% CI = 1.00 to 1.48]; PFS, HR = 1.27 [95% CI = 1.05 to 1.53]; postprogression survival, HR = 1.13 [95% CI = 0.90 to 1.43]). These trends were consistent with patients with right-sided *RAS* wild-type metastatic CRC (Figure 4, Figure S3).

### Correlations between depth of response and survival

**Figure 5.**
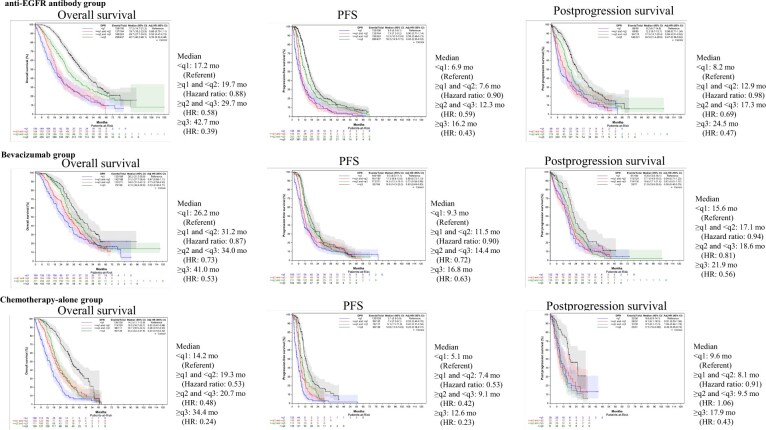
Correlations between depth of response and survival in left-sided tumors. This figure highlights the correlation between the depth of response and survival outcomes (overall survival, PFS, and postprogression survival) in left-sided tumors, stratified by treatment type (anti-EGFR antibody, bevacizumab, and chemotherapy alone). Patients with a greater depth of response experienced statistically significantly improved survival, with clear stratification across treatment groups. EGFR = epidermal growth factor receptor; HR = hazard ratio; PFS = progression-free survival; q = quantile.

To evaluate the significance of depth of response, we examined its implications in the left-sided anti-EGFR antibody group, the bevacizumab group, and chemotherapy-alone group, referencing the hazard ratio of each quantile of the maximum percentage reduction to the first quantile (q). In the anti-EGFR antibody group, the hazard ratios for overall survival were as follows: ≥q1 and <q2, 0.88 (95% CI = 0.70 to 1.11); ≥q2 and <q3, 0.58 (95% CI = 0.47 to 0.72); and ≥q3, 0.39 (95% CI = 0.32 to 0.48) compared with <q1. For PFS, the hazard ratios were 0.90 (95% CI = 0.71 to 1.14), 0.59 (95% CI = 0.48 to 0.73), and 0.43 (95% CI = 0.35 to 0.52). For postprogression survival, the hazard ratios were 0.98 (95% CI = 0.71 to 1.34), 0.69 (95% CI = 0.51 to 0.93), and 0.47 (95% CI = 0.36 to 0.62). In the bevacizumab group, the hazard ratios for overall survival were as follows: ≥q1 and <q2, 0.87 (95% CI = 0.68 to 1.11); ≥q2 and <q3, 0.73 (95% CI = 0.58 to 0.93); and ≥q4, 0.53 (95% CI = 0.40 to 0.71). The hazard ratios for PFS were 0.90 (95% CI = 0.72 to 1.13), 0.72 (95% CI = 0.58 to 0.90), and 0.63 (95% CI = 0.49 to 0.83). For postprogression survival, the hazard ratios were 0.94 (95% CI = 0.71 to 1.25), 0.81 (95% CI = 0.61 to 1.07), and 0.56 (95% CI = 0.40 to 0.79). In the chemotherapy-alone group, the hazard ratios for overall survival were as follows: ≥q1 and <q2, 0.53 (95% CI = 0.41 to 0.68); ≥q2 and <q3, 0.48 (95% CI = 0.37 to 0.63); and ≥q4, 0.24 (95% CI = 0.18 to 0.32). The hazard ratios for PFS were 0.53 (95% CI = 0.40 to 0.70), 0.42 (95% CI = 0.31 to 0.56), and 0.23 (95% CI = 0.18 to 0.31). For postprogression survival, the hazard ratios were 0.91 (95% CI = 0.53 to 1.56), 1.06 (95% CI = 0.64 to 1.74), and 0.43 (95% CI = 0.25 to 0.74). In patients with left-sided *RAS* wild-type metastatic CRC, depth of response was demonstrated to be a prognostic marker for overall survival, PFS, and postprogression survival, with survival curves showing clear stratification based on depth of response. These trends were consistent with patients with right-sided *RAS* wild-type metastatic CRC (Figure 5, Figure S4).

Next, we calculated the depth of response required to achieve the median overall survival in patients treated with chemotherapy combined with an anti-EGFR antibody and patients treated with chemotherapy combined with bevacizumab. In patients with left-sided disease receiving anti-EGFR–based therapy, the receiver operating characteristic curve–derived cutoff value to reach a median overall survival of 31.9 months was 0.55 (area under the curve = 0.70, sensitivity = 0.63, specificity = 0.68). For patients receiving bevacizumab-based therapy, the cutoff value to achieve a median overall survival of 32.7 months was 0.47 (area under the curve = 0.66, sensitivity = 0.73, specificity = 0.52) (Figure S5). These cutoff values for depth of response, as identified by the receiver operating characteristic curve, effectively stratified patients with improved overall survival, PFS, and postprogression survival. Furthermore, patients treated with chemotherapy plus an anti-EGFR antibody exhibited a trend toward better hazard ratios for overall survival, PFS, and postprogression survival compared with patients treated with chemotherapy plus bevacizumab or chemotherapy alone (Figure S5).

## Discussion

Although several literature-based meta-analyses have been published on the clinical efficacy of early tumor shrinkage and depth of response in patients with metastatic CRC,^13^^,^^24^ to the best of our knowledge, this study represents the largest investigation to date using individual patient data to examine the association between early tumor shrinkage and depth of response with overall survival, PFS, and postprogression survival in the context of chemotherapy combined with anti-EGFR antibodies or bevacizumab, according to tumor sidedness in patients with *RAS* wild-type metastatic CRC. In addition, our analysis encompasses evaluations conducted using various criteria, including World Health Organization and RECIST, versions 1.0 and 1.1, reflecting changes over time. The World Health Organization criteria allow for a maximum of 2 target lesions per organ and 5 target lesions in total, whereas RECIST, version 1.0, permits up to 5 target lesions per organ and 10 target lesions in total. RECIST, version 1.1, however, revised these criteria to allow a maximum of 2 target lesions per organ and 5 target lesions in total. In our analysis, we used the longest diameter measurements and demonstrated that early tumor shrinkage and depth of response consistently serve as reliable assessment metrics, maintaining stable values regardless of the number of tumor lesions, total tumor diameter, or the presence of liver metastases. Given the ability of early tumor shrinkage and depth of response to predict survival outcomes without incurring additional costs for data collection, we believe that these metrics should be actively used not only in clinical trials but also in routine clinical practice.

Although early tumor shrinkage and depth of response were shown to be strongly associated with survival outcomes, irrespective of the type of targeted therapy used or the primary tumor location, the findings were particularly pronounced in patients treated with chemotherapy plus an anti-EGFR antibody. These results suggest that a higher rate of early tumor shrinkage and a deeper depth of response are associated with statistically significantly better survival. Notably, in ultraselect patients with right-sided tumors characterized by specific gene alterations (*RAS*/*BRAF*/*EGFR* ectodomain wild type), early tumor shrinkage and depth of response may serve as prognostic markers for chemotherapy plus anti-EGFR antibody treatment. In our dataset, among patients with right-sided, *RAS/BRAF* wild-type disease treated chemotherapy plus anti-EGFR antibodies, the hazard ratios for early tumor shrinkage positivity and for depth of response in the highest quantile (≥Q3) were more favorable than those observed in patients treated with bevacizumab or chemotherapy alone (Table S5). Furthermore, although the current standard treatment for *RAS* wild-type left-sided metastatic CRC is chemotherapy plus an anti-EGFR antibody, our analysis revealed that patients who did not achieve early tumor shrinkage had worse overall survival, PFS, and postprogression survival compared with patients treated with chemotherapy plus bevacizumab. The ability to determine at the 8-week mark whether anti-EGFR antibody–based therapy may be unsuitable and potentially associated with shorter overall survival is crucial for making informed decisions regarding the continuation of treatment, independent of gene alterations. Although the present analysis alone does not allow us to conclude whether switching to bevacizumab-based therapy at the 8-week mark is appropriate, the possibility of a shorter PFS should be anticipated, and clinicians should be prepared to promptly transition to the next line of treatment, if necessary.

It was also demonstrated that depth of response, irrespective of the type of targeted therapy or the primary tumor location, serves as a robust indicator capable of effectively stratifying patient survival outcomes. Although the sensitivity and specificity of depth of response as a predictive marker may not be exceptionally high, our analysis revealed that the depth of response required to achieve the median overall survival was 55% for anti-EGFR antibody–based therapy and 47% for bevacizumab-based therapy (Figure S5). For both anti-EGFR antibody–based therapy and bevacizumab-based therapy, the area under the curve values were not particularly high, making it difficult to consider the model as ideal. However, considering the balance between sensitivity and specificity, it may serve as a useful indicator. In addition, achieving a deep response during first-line treatment can be considered a major factor influencing subsequent survival duration.

This study had several limitations. First, *BRAF* mutation status was unavailable in more than half the cases, and microsatellite instability status was missing in the majority of patients, precluding evaluation of their impact on survival outcomes. Second, due to the lack of information about the organs assessed as target lesions, we were unable to analyze the influence of metastatic sites on early tumor shrinkage and depth of response. Furthermore, information about second-line and later-line therapies was incomplete due to the follow-up framework of the clinical trials, limiting our ability to assess their impact on overall survival. Although this study represents the most precise meta-analysis to date using individual patient data, limitations remain in fully evaluating the impact of early tumor shrinkage and depth of response on survival. The interpretation of these results must consider recent advances in treatment that have expanded subsequent therapeutic options, and the fact that this analysis is based on a select population from prospective randomized controlled trials focusing on patients with *RAS* wild-type disease facilitated by improved diagnostic accuracy. Nevertheless, the ability to obtain a prognostic indicator without incurring additional costs is valuable.

In conclusion, this study is the largest to date using individual patient data to assess early tumor shrinkage and depth of response as prognostic markers in metastatic CRC. Our findings confirm that early tumor shrinkage and depth of response are robust indicators of overall survival, PFS, and postprogression survival, regardless of targeted therapy or tumor location. Notably, patients who did not achieve early tumor shrinkage had worse outcomes with anti-EGFR therapy than with bevacizumab. These results suggest that early tumor shrinkage and depth of response are valuable tools for guiding treatment strategies and should be integrated into clinical trials and routine practice to optimize patient outcomes.

## Supplementary Material

pkaf042_Supplementary_Data

## Data Availability

The data sharing of individual patient data from each participating trial will be subject to the policy and procedures of the institutions and groups that conducted the original study. The availability of integrated individual patient data in ARCAD is determined by the ARCAD Steering Committee based on research proposals submitted by qualified investigators. Access is granted exclusively for academic research purposes.

## References

[1] Sung H , Ferlay J, Siegel RL, et al Global cancer statistics 2020: GLOBOCAN estimates of incidence and mortality worldwide for 36 cancers in 185 countries. CA Cancer J Clin. 2021;71:209-249. 10.3322/caac.21660 33538338

[2] Cervantes A , Adam R, Roselló S, et al ESMO Guidelines Committee. Electronic address: Clinicalguidelines@esmo.org. Metastatic colorectal cancer: ESMO Clinical Practice Guideline for diagnosis, treatment and follow-up. Ann Oncol. 2023;34:10-32. 10.1016/j.annonc.2022.10.003 36307056

[3] Yoshino T , Arnold D, Taniguchi H, et al Pan-Asian adapted ESMO consensus guidelines for the management of patients with metastatic colorectal cancer: a JSMO-ESMO initiative endorsed by CSCO, KACO, MOS, SSO and TOS. Ann Oncol. 2018;29:44-70. 29155929 10.1093/annonc/mdx738

[4] Morris VK , Kennedy EB, Baxter NN, et al Treatment of metastatic colorectal cancer: ASCO guideline. J Clin Oncol. 2023;41:678-700. 10.1200/JCO.22.01690 36252154 PMC10506310

[5] Arnold D , Lueza B, Douillard JY, et al Prognostic and predictive value of primary tumour side in patients with RAS wild-type metastatic colorectal cancer treated with chemotherapy and EGFR directed antibodies in six randomized trials. Ann Oncol. 2017;28:1713-1729. 10.1093/annonc/mdx175 28407110 PMC6246616

[6] Watanabe J , Muro K, Shitara K, et al Panitumumab vs bevacizumab added to standard first-line chemotherapy and overall survival among patients with RAS wild-type, left-sided metastatic colorectal cancer: a randomized clinical trial. [published correction appears in JAMA. 2023 Jun 27; 329(24):2196. doi:10.1001/jama.2023.10533]. JAMA. 2023;329:1271-1282. 10.1001/jama.2023.4428 37071094 PMC10114040

[7] Van Cutsem E , Cervantes A, Adam R, et al ESMO consensus guidelines for the management of patients with metastatic colorectal cancer. Ann Oncol. 2016;27:1386-1422. 10.1093/annonc/mdw235 27380959

[8] Venook AP , Niedzwiecki D, Lenz HJ, et al Effect of first-line chemotherapy combined with cetuximab or bevacizumab on overall survival in patients with KRAS wild-type advanced or metastatic colorectal cancer: a randomized clinical trial. JAMA. 2017;317:2392-2401. 10.1001/jama.2017.7105 28632865 PMC5545896

[9] Stintzing S , Modest DP, Rossius L, et al; FIRE-3 Investigators. FOLFIRI plus cetuximab versus FOLFIRI plus bevacizumab for metastatic colorectal cancer (FIRE-3): a post-hoc analysis of tumour dynamics in the final RAS wild-type subgroup of this randomised open-label phase 3 trial. Lancet Oncol. 2016;17:e420. 10.1016/S1470-2045(16)30440-5 [published correction appears in *Lancet Oncol*. 2016;17:e479. 10.1016/S1470-2045(16)30514-9]. *Lancet Oncol*. 2016;17:1426-1434. 10.1016/S1470-2045(16)30269-8 27575024

[10] Piessevaux H , Buyse M, Schlichting M, et al Use of early tumor shrinkage to predict long-term outcome in metastatic colorectal cancer treated with cetuximab. J Clin Oncol. 2013;31:3764-3775. 10.1200/JCO.2012.42.8532 24043732

[11] Cremolini C , Loupakis F, Antoniotti C, et al Early tumor shrinkage and depth of response predict long-term outcome in metastatic colorectal cancer patients treated with first-line chemotherapy plus bevacizumab: results from phase III TRIBE trial by the Gruppo Oncologico del Nord Ovest. Ann Oncol. 2015;26:1188-1194. 10.1093/annonc/mdv112 25712456

[12] Heinemann V , Stintzing S, Modest DP, Giessen-Jung C, Michl M, Mansmann UR. Early tumour shrinkage (ETS) and depth of response (DpR) in the treatment of patients with metastatic colorectal cancer (mCRC). Eur J Cancer. 2015;51:1927-1936. 10.1016/j.ejca.2015.06.116 26188850

[13] Petrelli F , Pietrantonio F, Cremolini C, et al Early tumour shrinkage as a prognostic factor and surrogate end-point in colorectal cancer: a systematic review and pooled-analysis. Eur J Cancer. 2015;51:800-807. 10.1016/j.ejca.2015.02.011 25794604

[14] Taieb J , Rivera F, Siena S, et al Exploratory analyses assessing the impact of early tumour shrinkage and depth of response on survival outcomes in patients with RAS wild-type metastatic colorectal cancer receiving treatment in three randomised panitumumab trials. [published correction appears in J Cancer Res Clin Oncol. 2018 Apr; 144(4):795-797. doi:10.1007/s00432-018-2591-y]. J Cancer Res Clin Oncol. 2018;144:321-335. 10.1007/s00432-017-2534-z 29080924 PMC5794806

[15] Hofmann FO , Heinemann V, D’Anastasi M, et al Standard diametric versus volumetric early tumor shrinkage as a predictor of survival in metastatic colorectal cancer: Subgroup findings of the randomized, open-label phase III trial FIRE-3/AIO KRK-0306. Eur Radiol,. 2023;33:1174-1184. 10.1007/s00330-022-09053-2 35976398 PMC9889429

[16] Burzykowski T , Coart E, Saad ED, et al; Aide et Recherche en Cancerologie Digestive Group. Evaluation of continuous tumor-size-based end points as surrogates for overall survival in randomized clinical trials in metastatic colorectal cancer. JAMA Netw Open. 2019;2:e1911750. 10.1001/jamanetworkopen.2019.11750 31539075 PMC6755539

[17] Kurreck A , Geissler M, Martens UM, et al Dynamics in treatment response and disease progression of metastatic colorectal cancer (mCRC) patients with focus on BRAF status and primary tumor location: analysis of untreated RAS-wild-type mCRC patients receiving FOLFOXIRI either with or without panitumumab in the VOLFI trial (AIO KRK0109). J Cancer Res Clin Oncol. 2020;146:2681-2691. 32449003 10.1007/s00432-020-03257-z PMC7467910

[18] Sommerhäuser G , Kurreck A, Beck A, et al Depth of response of induction therapy and consecutive maintenance treatment in patients with RAS wild-type metastatic colorectal cancer: An analysis of the PanaMa trial (AIO KRK 0212). Eur J Cancer. 2023;178:37-48. 10.1016/j.ejca.2022.09.011 36399909

[19] de Gramont A , Haller DG, Sargent DJ, Tabernero J, Matheson A, Schilsky RL. Toward efficient trials in colorectal cancer: the ARCAD Clinical Trials Program. J Clin Oncol. 2010;28:527-530. 10.1200/JCO.2009.25.2544 19841311

[20] Takeda Y , Misumi T, Bando H, et al ARCAD-Asia initiative: Leveraging yesterday’s data for tomorrow. ESMO Gastrointestinal Oncology. 2023;2:100007-100006. 10.1016/j.esmogo.2023.08.006 PMC12836648 41647732

[21] Choi JH , Ahn MJ, Rhim HC, et al Comparison of WHO and RECIST criteria for response in metastatic colorectal carcinoma. Cancer Res Treat. 2005;37:290-293. 10.4143/crt.2005.37.5.290 19956529 PMC2785927

[22] Therasse P , Arbuck SG, Eisenhauer EA, et al New guidelines to evaluate the response to treatment in solid tumors. European Organization for Research and Treatment of Cancer, National Cancer Institute of the United States, National Cancer Institute of Canada. J Natl Cancer Inst. 2000;92:205-216. 10.1093/jnci/92.3.205 10655437

[23] Eisenhauer EA , Therasse P, Bogaerts J, et al New response evaluation criteria in solid tumours: Revised RECIST guideline (version 1.1). Eur J Cancer. 2009;45:228-247. 10.1016/j.ejca.2008.10.026 19097774

[24] Yoshino T , Hooda N, Younan D, et al A meta-analysis of efficacy and safety data from head-to-head first-line trials of epidermal growth factor receptor inhibitors versus bevacizumab in adult patients with RAS wild-type metastatic colorectal cancer by sidedness. Eur J Cancer. 2024;202:113975. 10.1016/j.ejca.2024.113975 38442645

