# Supplementary material for: Associations between early tumor shrinkage/depth of response and survival from the ARCAD database
Source: JNCI Cancer Spectr. 2025 Apr 25;9(3):pkaf042. doi: 10.1093/jncics/pkaf042 (PMC12159729; doi:10.1093/jncics/pkaf042)
Supplement: pkaf042_Supplementary_Data [file pkaf042_supplementary_data.docx]

**Supplementary Materials**

**Associations between Early Tumor Shrinkage/Depth of Response and Survivals from the ARCAD database**

**Supplementary Tables**

**Supplementary Table 1 Patient background (Left-sided)**

|  | Treatment | | |
| --- | --- | --- | --- |
|  | anti-EGFR antibody  (N=1075) | Bevacizumab  (N=693) | Chemotherapy alone (N=511) |
| **Performance Status**, n (%) |  |  |  |
| 0 | 704 (65.5%) | 507 (73.2%) | 268 (52.5%) |
| 1 | 352 (32.7%) | 185 (26.7%) | 215 (42.2%) |
| 2 | 19 (1.8%) | 1 (0.1%) | 27 (5.3%) |
| Missing | 0 | 0 | 1 |
|  |  |  |  |
| **Sex**, n (%) |  |  |  |
| Female | 364 (33.9%) | 229 (33.0%) | 189 (37.0%) |
| Male | 711 (66.1%) | 464 (67.0%) | 322 (63.0%) |
|  |  |  |  |
| **Age at enrollment** |  |  |  |
| N | 1075 | 693 | 511 |
| Mean (SD) | 60.3 (10.83) | 60.3 (10.88) | 59.2 (10.63) |
| Median | 62.0 | 62.0 | 60.0 |
| Range | 20.0, 81.0 | 21.0, 82.0 | 19.0, 84.0 |
|  |  |  |  |
| **Age at enrollment**, n (%) |  |  |  |
| <65 | 654 (60.8%) | 417 (60.2%) | 338 (66.1%) |
| ≥65 | 421 (39.2%) | 276 (39.8%) | 173 (33.9%) |
|  |  |  |  |
| **Carcinoembryonic Antigen (ng/mL)** |  |  |  |
| N | 504 | 416 | 204 |
| Mean (SD) | 641.4 (3617.43) | 499.5 (1662.47) | 446.2 (1165.39) |
| Median | 33.6 | 38.8 | 43.4 |
| Range | 0.0, 71000.0 | 0.8, 15000.0 | 0.5, 7550.0 |
|  |  |  |  |
| **Sum of Baseline Tumor Lesions (mm)** |  |  |  |
| N | 1073 | 692 | 511 |
| Mean (SD) | 111.1 (89.41) | 98.9 (75.30) | 139.4 (97.48) |
| Median | 87.0 | 80.2 | 120.0 |
| Range | 10.0, 560.0 | 6.0, 530.0 | 10.0, 624.0 |
|  |  |  |  |
| **Liver Metastasis**, n (%) |  |  |  |
| No Involvement | 132 (21.8%) | 139 (21.4%) | 0 (.%) |
| Liver Involvement Only | 255 (42.1%) | 276 (42.5%) | 0 (.%) |
| Liver and ≥ 1 non-Liver Involvement | 219 (36.1%) | 235 (36.2%) | 0 (.%) |
| Missing | 469 | 43 | 511 |
|  |  |  |  |
| **Peritoneal Metastasis**, n (%) |  |  |  |
| No Involvement | 280 (90.9%) | 332 (90.0%) | 0 (.%) |
| Peritoneal Involvement Only | 4 (1.3%) | 9 (2.4%) | 0 (.%) |
| Peritoneal and ≥ 1 non-Peritoneal Involvement | 24 (7.8%) | 28 (7.6%) | 0 (.%) |
| Missing | 767 | 324 | 511 |
|  |  |  |  |
| **N of Metastatic Sites**, n (%) |  |  |  |
| 0 | 6 (0.9%) | 5 (0.7%) | 0 (0.0%) |
| 1 | 377 (56.4%) | 385 (56.1%) | 34 (49.3%) |
| 2+ | 286 (42.8%) | 296 (43.1%) | 35 (50.7%) |
| Missing | 406 | 7 | 442 |
|  |  |  |  |
| **BRAF Mutation Status**, n (%) |  |  |  |
| MT | 23 (4.1%) | 22 (12.4%) | 20 (4.2%) |
| WT | 544 (95.9%) | 155 (87.6%) | 460 (95.8%) |
| Missing | 508 | 516 | 31 |
|  |  |  |  |
| **Prior Surgery (Any; Yes vs No)**, n (%) |  |  |  |
| No | 1 (0.2%) | 3 (1.9%) | 0 (0.0%) |
| Yes | 407 (99.8%) | 153 (98.1%) | 414 (100.0%) |
| Missing | 667 | 537 | 97 |
|  |  |  |  |
| **Sidedness**, n (%) |  |  |  |
| Left | 1075 (100.0%) | 693 (100.0%) | 511 (100.0%) |

EGFR, epidermal growth factor;

**Supplementary Table 2 Patient background (Right sided)**

|  | Treatment | | |
| --- | --- | --- | --- |
|  | anti-EGFR antibody (N=296) | Bevacizumab (N=239) | Chemotherapy alone (N=143) |
| **Performance Status**, n (%) |  |  |  |
| 0 | 187 (63.2%) | 159 (66.5%) | 86 (60.1%) |
| 1 | 106 (35.8%) | 79 (33.1%) | 52 (36.4%) |
| 2 | 3 (1.0%) | 1 (0.4%) | 5 (3.5%) |
|  |  |  |  |
| **Sex**, n (%) |  |  |  |
| Female | 150 (50.7%) | 110 (46.0%) | 71 (49.7%) |
| Male | 146 (49.3%) | 129 (54.0%) | 72 (50.3%) |
|  |  |  |  |
| **Age at enrollment** |  |  |  |
| N | 296 | 239 | 143 |
| Mean (SD) | 62.6 (10.77) | 61.6 (11.41) | 59.9 (12.36) |
| Median | 64.0 | 64.0 | 62.0 |
| Range | 22.0, 89.0 | 23.0, 83.0 | 24.0, 82.0 |
|  |  |  |  |
| **Age at enrollment**, n (%) |  |  |  |
| <65 | 155 (52.4%) | 123 (51.5%) | 87 (60.8%) |
| ≥65 | 141 (47.6%) | 116 (48.5%) | 56 (39.2%) |
|  |  |  |  |
| **Carcinoembryonic Antigen (ng/mL)** |  |  |  |
| N | 126 | 109 | 57 |
| Mean (SD) | 429.2 (1638.64) | 351.4 (1019.67) | 130.3 (492.87) |
| Median | 20.6 | 18.3 | 15.0 |
| Range | 0.8, 14687.0 | 0.2, 5440.9 | 0.6, 3592.0 |
|  |  |  |  |
| **Sum of Baseline T-lesions (mm)** |  |  |  |
| N | 296 | 239 | 143 |
| Mean (SD) | 108.3 (90.00) | 85.9 (62.10) | 104.2 (71.49) |
| Median | 85.5 | 73.0 | 91.0 |
| Range | 10.0, 574.0 | 10.0, 378.0 | 13.0, 370.0 |
|  |  |  |  |
| **Liver Metastasis**, n (%) |  |  |  |
| No Involvement | 50 (26.9%) | 69 (29.0%) | 0 (.%) |
| Liver Involvement Only | 65 (34.9%) | 79 (33.2%) | 0 (.%) |
| Liver and ≥ 1 non-Liver Involvement | 71 (38.2%) | 90 (37.8%) | 0 (.%) |
| Missing | 110 | 1 | 143 |
|  |  |  |  |
| **Peritoneal Metastasis**, n (%) |  |  |  |
| 0 | 58 (74.4%) | 86 (77.5%) | 0 (.%) |
| 1 | 6 (7.7%) | 6 (5.4%) | 0 (.%) |
| 2 | 14 (17.9%) | 19 (17.1%) | 0 (.%) |
| Missing | 218 | 128 | 143 |
|  |  |  |  |
| **N of Metastatic Sites**, n (%) |  |  |  |
| 0 | 4 (2.0%) | 5 (2.1%) | 0 (0.0%) |
| 1 | 103 (52.6%) | 118 (49.6%) | 6 (35.3%) |
| 2+ | 89 (45.4%) | 115 (48.3%) | 11 (64.7%) |
| Missing | 100 | 1 | 126 |
|  |  |  |  |
| **BRAF Mutation Status**, n (%) |  |  |  |
| MT | 44 (27.8%) | 35 (41.7%) | 32 (23.4%) |
| WT | 114 (72.2%) | 49 (58.3%) | 105 (76.6%) |
| Missing | 138 | 155 | 6 |
|  |  |  |  |
| **Prior Surgery (Any; Yes vs No)**, n (%) |  |  |  |
| No | 0 (0.0%) | 1 (4.0%) | 0 (0.0%) |
| Yes | 104 (100.0%) | 24 (96.0%) | 128 (100.0%) |
| Missing | 192 | 214 | 15 |
|  |  |  |  |
| **Sidedness**, n (%) |  |  |  |
| Right | 296 (100.0%) | 239 (100.0%) | 143 (100.0%) |

EGFR, epidermal growth factor;

**Supplementary Table 3. The Correlations between Early Tumor Shrinkage/Depth of Response and Survival With/Without Liver or Peritoneal Metastases (Left-sided)**

| **ETS/DPR** | **Metastases Site** | **Drug Group** | **Category** | **OS** | | | **PFS** | | | **PPS** | | |
| --- | --- | --- | --- | --- | --- | --- | --- | --- | --- | --- | --- | --- |
|  |  |  |  | **Events/Total** | **Median (months)** | **Hazard Ratio** | **Events/Total** | **Median (months)** | **Hazard Ratio** | **Events/Total** | **Median (months)** | **Hazard Ratio** |
| ETS | Without Liver Metastasis | Anti-EGFR antibody | ETS- | 25/31 | 24.0 | Ref | 31/31 | 8.0 | Ref | 25/31 | 12.9 | Ref |
|  |  |  | ETS+ | 50/64 | 41.6 | 0.44 | 64/64 | 13.6 | 0.44 | 50/64 | 24.4 | 0.50 |
|  |  | Bevacizumab | ETS- | 32/39 | 27.7 | Ref | 39/39 | 11.4 | Ref | 32/39 | 15.1 | Ref |
|  |  |  | ETS+ | 51/60 | 27.7 | 1.04 | 60/60 | 10.9 | 0.99 | 51/60 | 17.2 | 1.10 |
|  | With Liver Metastasis | Anti-EGFR antibody | ETS- | NA | NA | Ref | 83/83 | 7.4 | Ref | 73/83 | 12.8 | Ref |
|  |  |  | ETS+ | NA | NA | NA | 256/256 | 12.8 | 0.57 | 200/256 | 19.9 | 0.52 |
|  |  | Bevacizumab | ETS- | NA | NA | Ref | 133/133 | 9.3 | Ref | 125/133 | 16.3 | Ref |
|  |  |  | ETS+ | NA | NA | NA | 213/213 | 11.6 | 0.71 | 184/213 | 20.6 | 0.59 |
| DPR | Without Liver Metastasis | Anti-EGFR antibody | <q1 | 18/21 | 30.3 | Ref | 19/21 | 12.3 | Ref | 15/16 | 20.5 | Ref |
|  |  |  | ≥q1 and <q2 | 8/9 | 28.1 | 0.85 | 9/9 | 9.4 | 1.22 | 8/9 | 22.1 | 0.93 |
|  |  |  | ≥q2 and <q3 | 4/7 | 73.7 | 0.36 | 7/7 | 13.1 | 1.66 | 4/7 | 60.0 | 0.33 |
|  |  |  | ≥q3 | 8/13 | 44.7 | 0.39 | 11/13 | 21.7 | 0.50 | 6/9 | 34.4 | 0.36 |
|  |  | Bevacizumab | <q1 | 30/38 | 27.6 | Ref | 36/38 | 10.2 | Ref | 20/23 | 14.6 | Ref |
|  |  |  | ≥q1 and <q2 | 20/26 | 29.7 | 0.76 | 25/26 | 11.2 | 0.88 | 17/21 | 22.1 | 0.71 |
|  |  |  | ≥q2 and <q3 | 18/26 | 37.5 | 0.68 | 24/26 | 17.6 | 0.49 | 15/20 | 15.4 | 1.09 |
|  |  |  | ≥q3 | 11/15 | 39.4 | 0.62 | 14/15 | 11.1 | 0.62 | 9/12 | 26.1 | 0.71 |
|  | With Liver Metastasis | Anti-EGFR antibody | <q1 | 31/40 | 24.9 | Ref | 35/40 | 8.4 | Ref | 27/31 | 13.9 | Ref |
|  |  |  | ≥q1 and <q2 | 12/15 | 26.8 | 1.10 | 14/15 | 7.4 | 1.43 | 11/13 | 17.5 | 0.87 |
|  |  |  | ≥q2 and <q3 | 25/45 | 35.3 | 0.53 | 34/45 | 17.5 | 0.53 | 20/29 | 19.9 | 0.58 |
|  |  |  | ≥q3 | 39/73 | 51.1 | 0.38 | 57/73 | 17.7 | 0.53 | 38/56 | 34.0 | 0.43 |
|  |  | Bevacizumab | <q1 | 58/69 | 23.0 | Ref | 63/69 | 9.2 | Ref | 35/57 | 14.6 | Ref |
|  |  |  | ≥q1 and <q2 | 50/72 | 26.9 | 0.78 | 59/72 | 12.0 | 0.78 | 39/42 | 13.5 | 1.02 |
|  |  |  | ≥q2 and <q3 | 52/83 | 33.6 | 0.57 | 62/83 | 14.6 | 0.55 | 37/45 | 22.0 | 0.74 |
|  |  |  | ≥q3 | 25/41 | 41.0 | 0.44 | 35/41 | 15.9 | 0.57 | 20/24 | 22.8 | 0.57 |

ETS, Early Tumor Shrinkage; DpR, Depth of Response; EGFR, epidermal growth factor;

**Supplementary Table 4. The Correlations between Early Tumor Shrinkage/Depth of Response and Survival With/Without Liver (Right-sided)**

| **ETS/DPR** | **Metastases Site** | **Drug Group** | **Category** | **OS** | | | **PFS** | | | **PPS** | | |
| --- | --- | --- | --- | --- | --- | --- | --- | --- | --- | --- | --- | --- |
|  |  |  |  | **Events/Total** | **Median (months)** | **Hazard Ratio** | **Events/Total** | **Median (months)** | **Hazard Ratio** | **Events/Total** | **Median (months)** | **Hazard Ratio** |
| ETS | Without Liver Metastasis | Anti-EGFR antibody | ETS- | 19/20 | 15.8 | Ref | 20/20 | 6.8 | Ref | 19/20 | 6.9 | Ref |
|  |  |  | ETS+ | 22/30 | 32.0 | 0.33 | 27/30 | 13.6 | 0.56 | 22/30 | 18.0 | 0.45 |
|  |  | Bevacizumab | ETS- | 30/32 | 27.1 | Ref | 30/32 | 7.5 | Ref | 30/32 | 11.2 | Ref |
|  |  |  | ETS+ | 31/37 | 21.9 | 0.60 | 34.37 | 11.1 | 0.47 | 31/37 | 6.9 | 0.90 |
|  | With Liver Metastasis | Anti-EGFR antibody | ETS- | 40/47 | 14.5 | Ref | 45/47 | 7.1 | Ref | 40/47 | 7.9 | Ref |
|  |  |  | ETS+ | 72/89 | 26.5 | 0.60 | 80/89 | 10.1 | 0.62 | 72/89 | 13.4 | 0.65 |
|  |  | Bevacizumab | ETS- | 71/79 | 15.1 | Ref | 77/79 | 7.1 | Ref | 71/79 | 7.2 | Ref |
|  |  |  | ETS+ | 69/90 | 28.4 | 0.47 | 77/90 | 11.4 | 0.49 | 69/90 | 16.2 | 0.53 |
| DPR | Without Liver Metastasis | Anti-EGFR antibody | <q1 | 19/19 | 10.7 | Ref | 19/19 | 5.7 | Ref | 15/15 | 3.3 | Ref |
|  |  |  | ≥q1 and <q2 | 2/2 | 6.7 | 2.41 | 2/2 | 6.2 | 1.96 | 1/1 | 1.0 | 6.79 |
|  |  |  | ≥q2 and <q3 | 2/4 | NE | 0.21 | 3/4 | 10.6 | 0.23 | 1/2 | NE | 0.19 |
|  |  |  | ≥q3 | 3/5 | 54.4 | 0.32 | 4/5 | 18.2 | 0.16 | 2/3 | 36.2 | 0.49 |
|  |  | Bevacizumab | <q1 | 20/26 | 22.0 | Ref | 23/26 | 8.0 | Ref | 13/16 | 11.4 | Ref |
|  |  |  | ≥q1 and <q2 | 3/4 | 28.1 | 3.47 | 3/4 | 4.4 | 2.08 | 1/1 | 41.6 | 24.39 |
|  |  |  | ≥q2 and <q3 | 6/6 | 13.2 | 2.70 | 6/6 | 9.5 | 1.31 | 3/3 | 3.1 | 14.80 |
|  |  |  | ≥q3 | 2/4 | 82.0 | 0.42 | 3/4 | 28.4 | 0.40 | 2/3 | 54.2 | 0.46 |
|  | With Liver Metastasis | Anti-EGFR antibody | <q1 | 18/21 | 13.5 | Ref | 20/21 | 5.3 | Ref | 15/17 | 8.8 | Ref |
|  |  |  | ≥q1 and <q2 | 15/17 | 19.0 | 1.17 | 16/17 | 6.3 | 0.70 | 14/15 | 12.2 | 1.54 |
|  |  |  | ≥q2 and <q3 | 11/14 | 18.1 | 0.79 | 12/14 | 9.3 | 0.38 | 9/10 | 9.6 | 1.32 |
|  |  |  | ≥q3 | 13/23 | 52.1 | 0.42 | 18/23 | 15.4 | 0.30 | 13/18 | 29.0 | 0.64 |
|  |  | Bevacizumab | <q1 | 28/36 | 14.2 | Ref | 18/21 | 6.9 | Ref | 18/21 | 6.9 | Ref |
|  |  |  | ≥q1 and <q2 | 14/16 | 16.7 | 0.99 | 10/10 | 4.3 | 1.17 | 10/10 | 4.3 | 1.17 |
|  |  |  | ≥q2 and <q3 | 12/18 | 29.2 | 0.37 | 9/13 | 18.1 | 0.39 | 9/13 | 18.1 | 0.39 |
|  |  |  | ≥q3 | 12/15 | 29.0 | 0.43 | 12/13 | 15.3 | 0.43 | 12/13 | 15.3 | 0.43 |

ETS, Early Tumor Shrinkage; DpR, Depth of Response; EGFR, epidermal growth factor;

**Supplementary Table 5. The Correlations between Early Tumor Shrinkage/Depth of Response and Survival in Right-sided *RAS/BRAF* wild-type Patients**

| **ETS/DPR** | **Drug Group** | **Category** | **OS** | | | **PFS** | | | **PPS** | | |
| --- | --- | --- | --- | --- | --- | --- | --- | --- | --- | --- | --- |
|  |  |  | **Events/Total** | **Median (months)** | **Hazard Ratio** | **Events/Total** | **Median (months)** | **Hazard Ratio** | **Events/Total** | **Median (months)** | **Hazard Ratio** |
| ETS | Anti-EGFR antibody | ETS- | 38/44 | 14.5 | Ref | 40/44 | 6.0 | Ref | 38/44 | 8.8 | Ref |
|  |  | ETS+ | 56/70 | 24.9 | 0.49 | 50/70 | 11.3 | 0.49 | 56/70 | 14.1 | 0.53 |
|  | Bevacizumab | ETS- | 19/24 | 22.6 | Ref | 22/24 | 9.2 | Ref | 19/24 | 11.2 | Ref |
|  |  | ETS+ | 19/25 | 33.5 | 0.63 | 20/25 | 16.1 | 0.54 | 19/25 | 20.2 | 0.66 |
|  | Chemotherapy alone | ETS- | 37/49 | 17.1 | Ref | 33/49 | 7.6 | Ref | 37/49 | 10.3 | Ref |
|  |  | ETS+ | 41/56 | 21.2 | 0.92 | 38/56 | 9.2 | 0.75 | 41/56 | 10.0 | 0.97 |
| DPR | Anti-EGFR antibody | <q1 | 27/30 | 7.4 | Ref | 26/30 | 3.9 | Ref | 7/7 | 4.2 | Ref |
|  |  | ≥q1 and <q2 | 13/14 | 9.9 | 0.49 | 12/14 | 4.9 | 0.49 | 5/6 | 7.8 | 0.44 |
|  |  | ≥q2 and <q3 | 17/21 | 25.2 | 0.14 | 14/21 | 8.9 | 0.14 | 6/9 | 16.0 | 0.18 |
|  |  | ≥q3 | 17/26 | 40.2 | 0.09 | 14/26 | 12.3 | 0.07 | 7/8 | 8.0 | 0.91 |
|  | Bevacizumab | <q1 | 4/5 | 12.5 | Ref | 5/5 | 10.4 | Ref | NA | NA | Ref |
|  |  | ≥q1 and <q2 | 1/1 | 22.6 | 63.27 | 1/1 | 6.4 | 0.00 | NA | NA | NA |
|  |  | ≥q2 and <q3 | 1/1 | 16.2 | 0.00 | 1/1 | 14.5 | 0.00 | NA | NA | NA |
|  |  | ≥q3 | NA | NA | NA | NA | NA | NA | NA | NA | NA |
|  | Chemotherapy alone | <q1 | 27/30 | 10.4 | Ref | 23/30 | 3.8 | Ref | 7/7 | 4.7 | Ref |
|  |  | ≥q1 and <q2 | 23/30 | 16.2 | 0.52 | 20/30 | 7.4 | 0.39 | 7/8 | 5.6 | 0.67 |
|  |  | ≥q2 and <q3 | 18/25 | 21.6 | 0.53 | 16/25 | 9.7 | 0.26 | 3/6 | 18.8 | 0.20 |
|  |  | ≥q3 | 14/25 | 37.6 | 0.26 | 17/25 | 12.1 | 0.14 | 8/13 | 17.8 | 0.27 |

ETS, Early Tumor Shrinkage; DpR, Depth of Response; EGFR, epidermal growth factor;

**Supplementary Figures**

**Supplemental Figure 1. Correlations Between Depth of Response and Number of Baseline Targeted Lesions/Total Tumor Diameter**


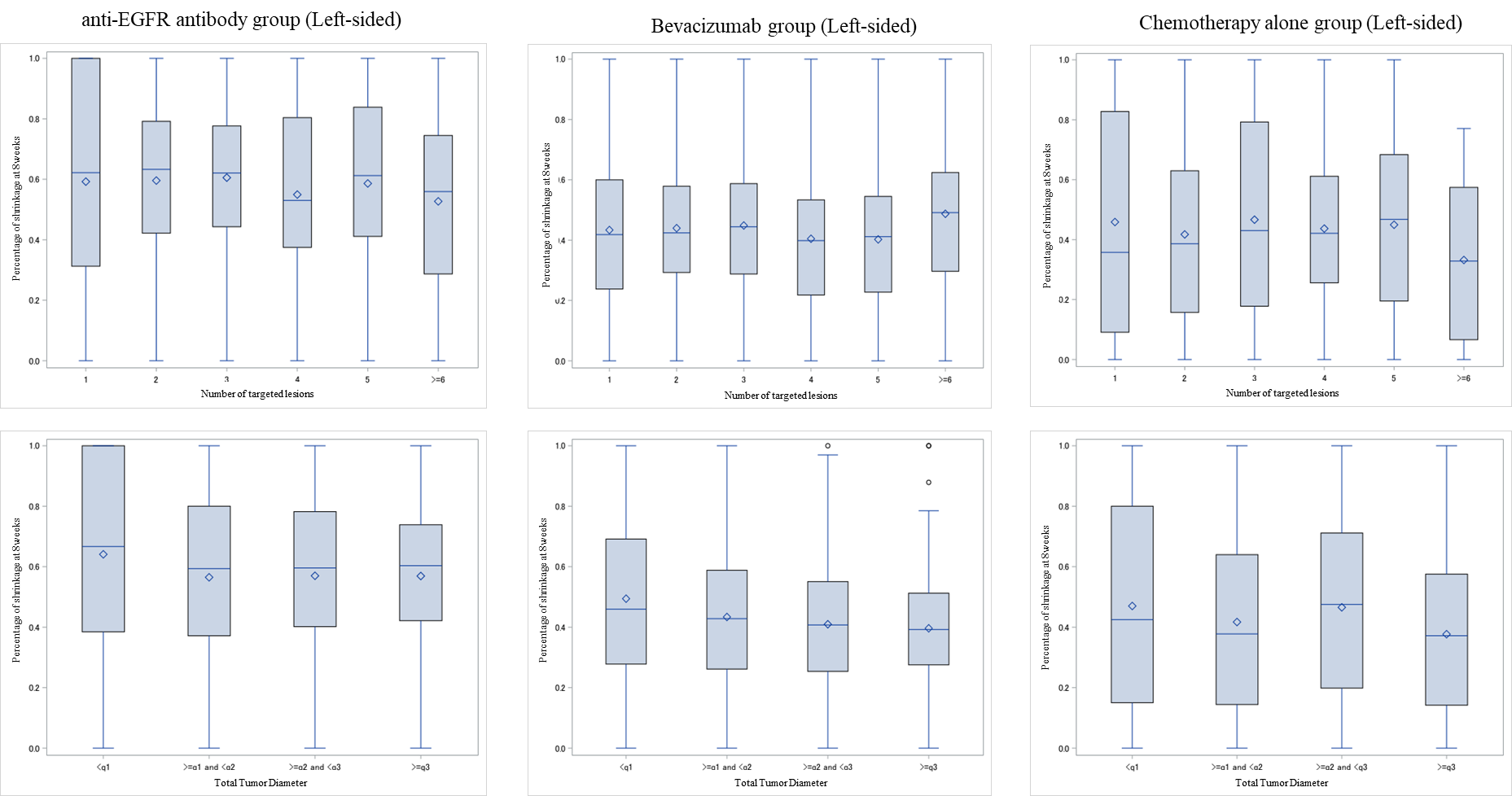


This figure shows the relationship between the depth of response (DpR) and the number of baseline target lesions or total tumor diameter. Data are presented for patients with left-sided *RAS* wild-type tumors across three treatment groups (anti-EGFR antibody, bevacizumab, and chemotherapy-alone), showing the percentage of shrinkage at 8 weeks.

**Supplemental Figure 2.** **Correlations Between Early Tumor Shrinkage and Survival in Right-Sided Tumors**


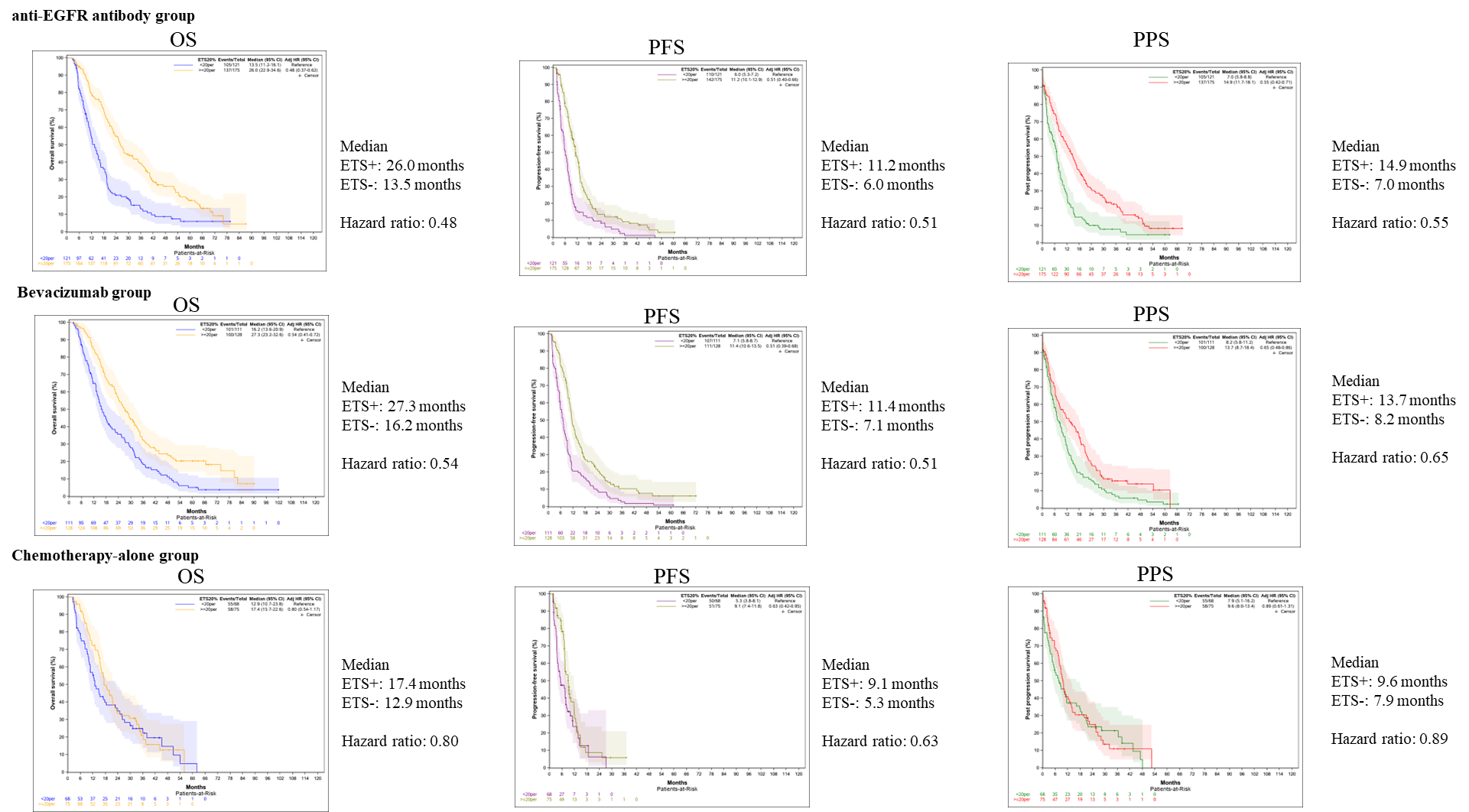


The figure displays the correlation between early tumor shrinkage (ETS) and survival outcomes (overall survival [OS], progression-free survival [PFS], and post-progression survival [PPS]) in right-sided *RAS* wild-type tumors. ETS-positive patients exhibited better survival outcomes compared to ETS-negative patients across all treatment groups (anti-EGFR antibody, bevacizumab, and chemotherapy-alone).

**Supplemental Figure 3.** **Correlations Between Early Tumor Shrinkage and Treatment Efficacy in Right-Sided Tumors**


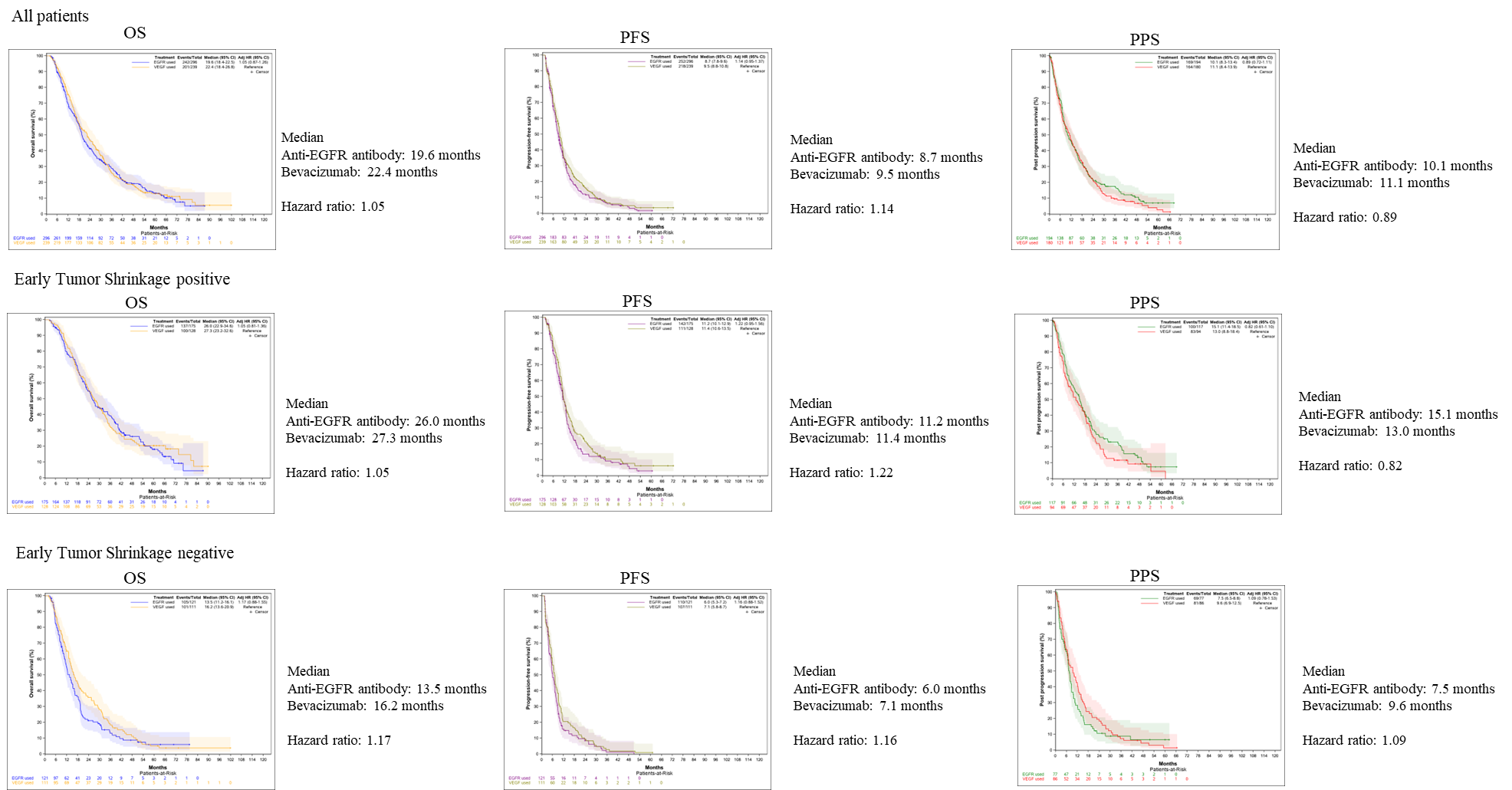


This figure compares the treatment efficacy of anti-EGFR antibody and bevacizumab in early tumor shrinkage (ETS)-positive and ETS-negative patients with right-sided tumors. The analysis showed that ETS-negative patients treated with bevacizumab demonstrated improved survival compared to those treated with anti-EGFR antibodies.

**Supplemental Figure 4.** **Correlations Between Depth of Response and Survival in Right-Sided Tumors**


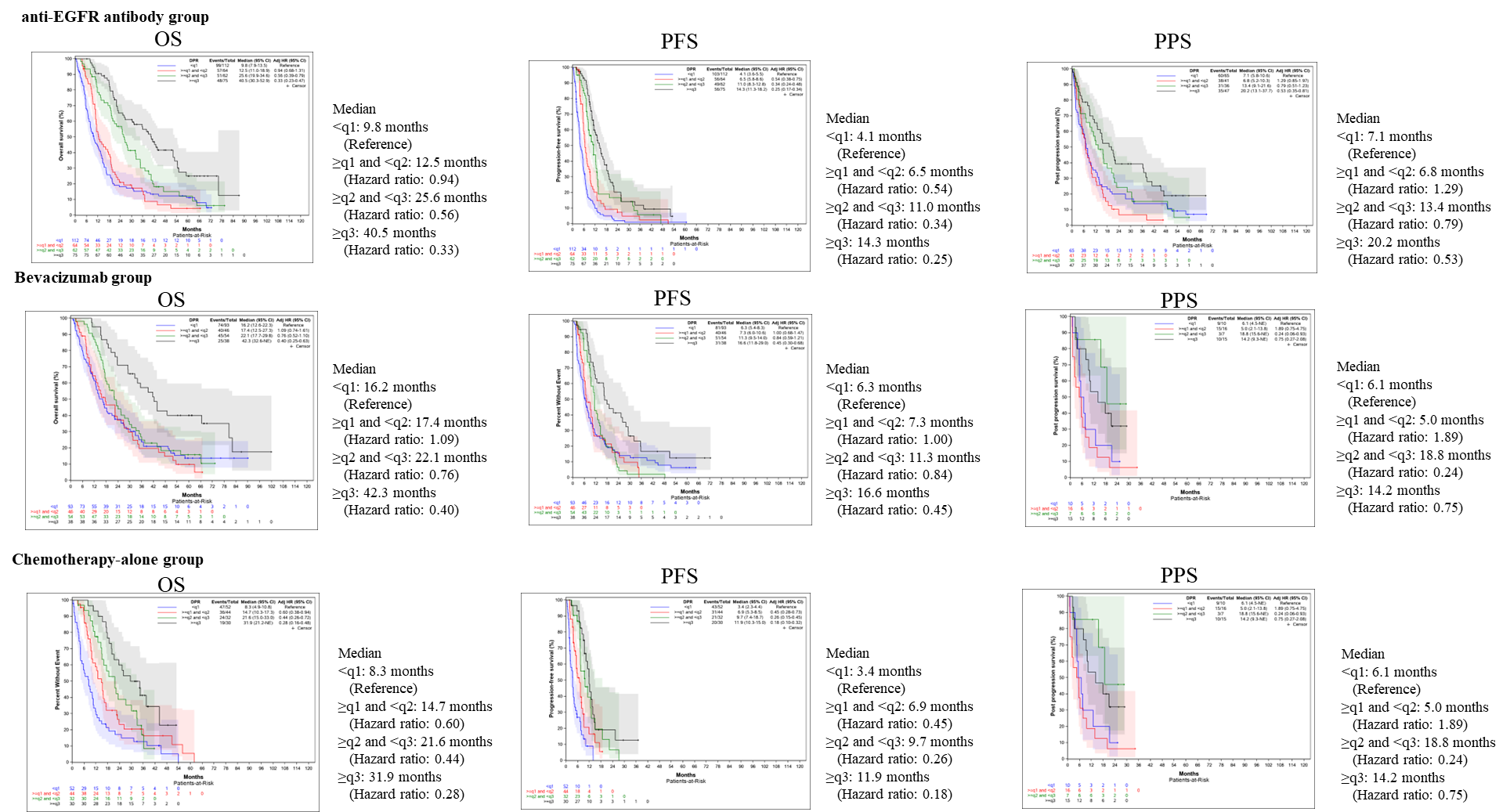


This figure illustrates the relationship between depth of response (DpR) and survival outcomes (overall survival [OS], progression-free survival [PFS], and post-progression survival [PPS]) in right-sided *RAS* wild-type tumors, stratified by treatment type (anti-EGFR antibody, bevacizumab, and chemotherapy-alone). Patients with a greater DpR exhibited better survival outcomes, with clear stratification across treatment groups.

**Supplemental Figure 5.** **ROC Curve for Required Depth of Response to Median Overall Survival in Left-Sided Tumors**


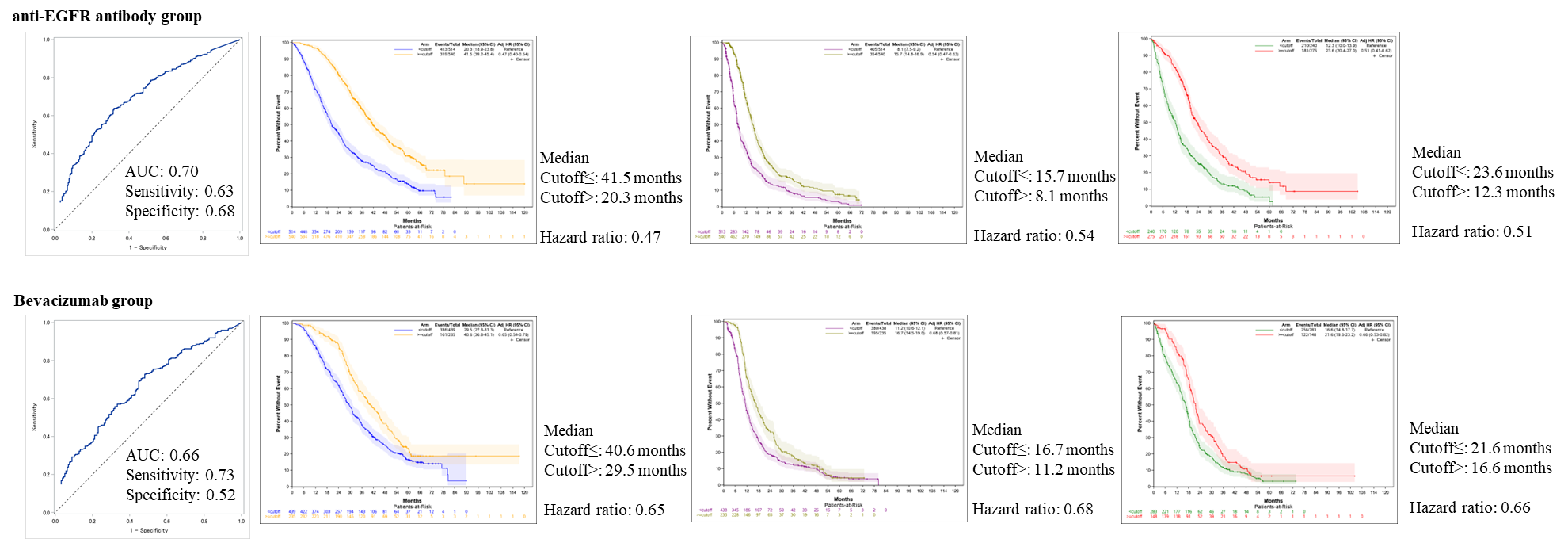


This figure presents the receiver operating characteristic (ROC) curve showing the depth of response (DpR) required to achieve the median overall survival in left-sided tumors treated with anti-EGFR antibody or bevacizumab. The figure provides cutoff values, sensitivity, and specificity for both treatments, demonstrating how a deeper response correlates with improved survival outcomes
